# Physical distancing and risk of COVID-19 in small-scale fisheries: a remote sensing assessment in coastal Ghana

**DOI:** 10.1038/s41598-020-79898-4

**Published:** 2020-12-29

**Authors:** Isaac Okyere, Ernest O. Chuku, Bernard Ekumah, Donatus B. Angnuureng, Justice K. Boakye-Appiah, David J. Mills, Raymond Babanawo, Noble K. Asare, Denis W. Aheto, Brian Crawford

**Affiliations:** 1 Department of Fisheries and Aquatic Sciences, University of Cape Coast, Cape Coast, Ghana; 2 Centre for Coastal Management (Africa Centre of Excellence in Coastal Resilience - ACECoR), University of Cape Coast, Cape Coast, Ghana; 3 Department of Environmental Science, University of Cape Coast, Cape Coast, Ghana; 4 Institute for Infection and Immunity, St George’s University of London/Hospital, Cranmer Terrace, London, SW17 0RE UK; 5 WorldFish, Bayan Lepas, Penang Malaysia; 6 ARC Centre of Excellence for Coral Reef Studies, James Cook University, Townsville, QLD Australia; 7 Coastal Resources Center, University of Rhode Island, Narragansett, RI USA

**Keywords:** Viral infection, Scientific data

## Abstract

The novel coronavirus is predicted to have dire implications on global food systems including fisheries value chains due to restrictions imposed on human movements in many countries. In Ghana, food production, both agriculture and fisheries, is exempted from restrictions as an essential service. The enforcement of COVID-19 prevention protocols, particularly social distancing, has been widely reported in Ghana’s agricultural markets whereas casual observations and media reports on fish landing sites suggest no such enforcements are in place. This study aimed to provide sound scientific evidence as a basis for informed policy direction and intervention for the artisanal fishing sector in these challenging times. We employed an unmanned aerial vehicle in assessing the risk of artisanal fishers to the pandemic using physical distancing as a proxy. From analysis of cumulative distribution function (G-function) of the nearest-neighbour distances, this study underscored crowding at all surveyed fish landing beaches, and identified potential “hotspots” for disease transmission. Aerial measurements taken at times of peak landing beach activity indicated that the highest proportion of people, representing 56%, 48%, 39% and 78% in Elmina, Winneba, Apam and Mumford respectively, were located at distances of less than one metre from their nearest neighbour. Risk of crowding was independent of the population at the landing beaches, suggesting that all categories of fish landing sites along the coast would require equal urgency and measured attention towards preventing and mitigating the spread of the disease.

## Introduction

The importance of small-scale fisheries to food and nutrition security and the livelihoods of many people within and beyond coastal areas of developing countries cannot be overemphasised. Globally, the small-scale fisheries sector is estimated to support 32 million direct jobs^1^. Over half of the catch in developing countries is produced by the small-scale fisheries sector, and 90 to 95 percent of the small-scale landings are used for local human consumption^1^. In Ghana, the fisheries sector provided an annual revenue of more than US$ 1 billion in 2015^2^. With substantially higher per capita consumption of fish (24 kg) in Ghana compared to the world average (16 kg), fish remains the principal source of low cost protein accounting for about 60% of animal protein in the local diet^3^, and contributes substantially to the supply of essential micro-nutrients^4^. As a dominant contributor to livelihoods in coastal areas, the fisheries sector provides direct and indirect employment for approximately 10% of the country’s population of 30 million people^2^. A majority of these are small-scale (referred to as artisanal) fishers operating from 186 fishing villages and nearly 300 landing beaches along the coast^5^. They contribute approximately 70% of the country’s marine fish production^2^. Typical of labour-intensive artisanal fisheries, fishing is characterised by clustering of fishers and close human interactions during harvesting while onboard vessels, offloading fish from canoes to landing beaches, at processing sites and in fish markets.

The end of 2019 saw the emergence of the severe acute respiratory syndrome virus 2 (SARS-CoV-2), the causative agent of COVID-19 in humans as reported by the World Health Organisation (WHO)^8^. By the second week of March 2020, the disease had so widely spread with rising incidence that the WHO declared it a pandemic emergency^6^. By its mode of spread, mainly through aerosols, physical distancing (otherwise called social distancing) between individuals remains one of the main public health measures to curb its spread. This has so far been the focus of efforts to control the disease and reduce infection rates^7^. The WHO therefore recommended a minimum distance of 1 m between individuals at any point in time and especially in public places^8^. Some countries however recommend approximately 2 m (6 feet) in their local guidelines^9^.

The COVID-19 pandemic was projected to have significant impacts on global fisheries systems throughout value chains^10,11^. Although SARS-CoV-2 is not known to infect or contaminate fish^12^, fishing communities are considered to be at high risk, serving as potential “hotspots” for rapid spread of the virus due to the migratory and huddling behaviour of fishers, and at times poor hygienic practices in these communities^10,11^. While implementing stringent restrictions on movement and physical contact to curb the spread of the disease, countries have also critically weighed the balance between disease control and nutritional needs, and most have allowed sectors within the national food system, including the fishing industry, to continue operating. Ghana, in its implementation of an emergency response to the COVID-19 pandemic, exempted fishers from a partial lockdown due to their essential role in food supply^13^. Despite being recognised “hotspots” for virus transmission, no targeted and coordinated measures have been instituted to control physical contact and ensure adherence to the protocols on social distancing and other preventive measures at fish landing beaches.

Fishing communities in Ghana are typically highly populated with poor sanitation practices^14^ and low literacy rates^5^. Given the communal nature of artisanal fishing, anticipating voluntary compliance from fishers of COVID-19 preventive measures which they may or may not be aware of is unrealistic. Of serious concern is a misconception that fishers are immune to the virus due to their proximity to the sea^15^. Extra effort will likely be needed to break the disease transmission chain and prevent escalation among local fishers should an outbreak occur in fishing communities.

Considering the rising number of COVID-19 cases in Ghana at the time of writing, and especially as cases have been recorded in all four coastal regions of the country by the Ghana Health Service^16^, it is important to rapidly assess the risk among different categories of fishing communities to the pandemic, and identify potential high risk areas (hotspots) to address rapid control measures. The outcome of the rapid assessments will assist government, development agencies and NGOs in developing targeted interventions to be instituted at high-risk landing beaches to reduce the risk to fishers, their families and the community.

To this end, this study aimed to assess the extent of clustering of fishers at different categories of small-scale fish landing beaches (low, moderate and highly populated) in order to identify potential “hotspots” for spread of the disease. The exigency of the pandemic required a swift survey within fishing communities for expedited intervention. Hence, we employed a rapid appraisal methodology using an Unmanned Aerial Vehicle (UAV) and Geographic Information System (GIS) tools. The aerial view not only provides the physical opportunity to assess distance between subjects with no or minimal intervention, but it does so quickly and at minimal risk to all involved, whilst effectively preserving anonymity of subjects.

## Materials and methods

### Study area and selection of landing beaches

The study was carried out at six landing beaches along the coast of the Central Region of Ghana. According to the 2016 Ghana Canoe Frame Survey^5^, the coastline of the Central Region is dotted with 97 landing beaches; the highest density among the four coastal regions in the country (the others are Western—89, Greater Accra—59 and Volta—47). Aside from its numerous landing beaches and vibrant artisanal fishery, the study was limited to the Central Region (location of the research team) due to COVID-19 lockdowns and other travel restrictions imposed on key regional corridors at the time of the study^13^.

**Figure 1:**
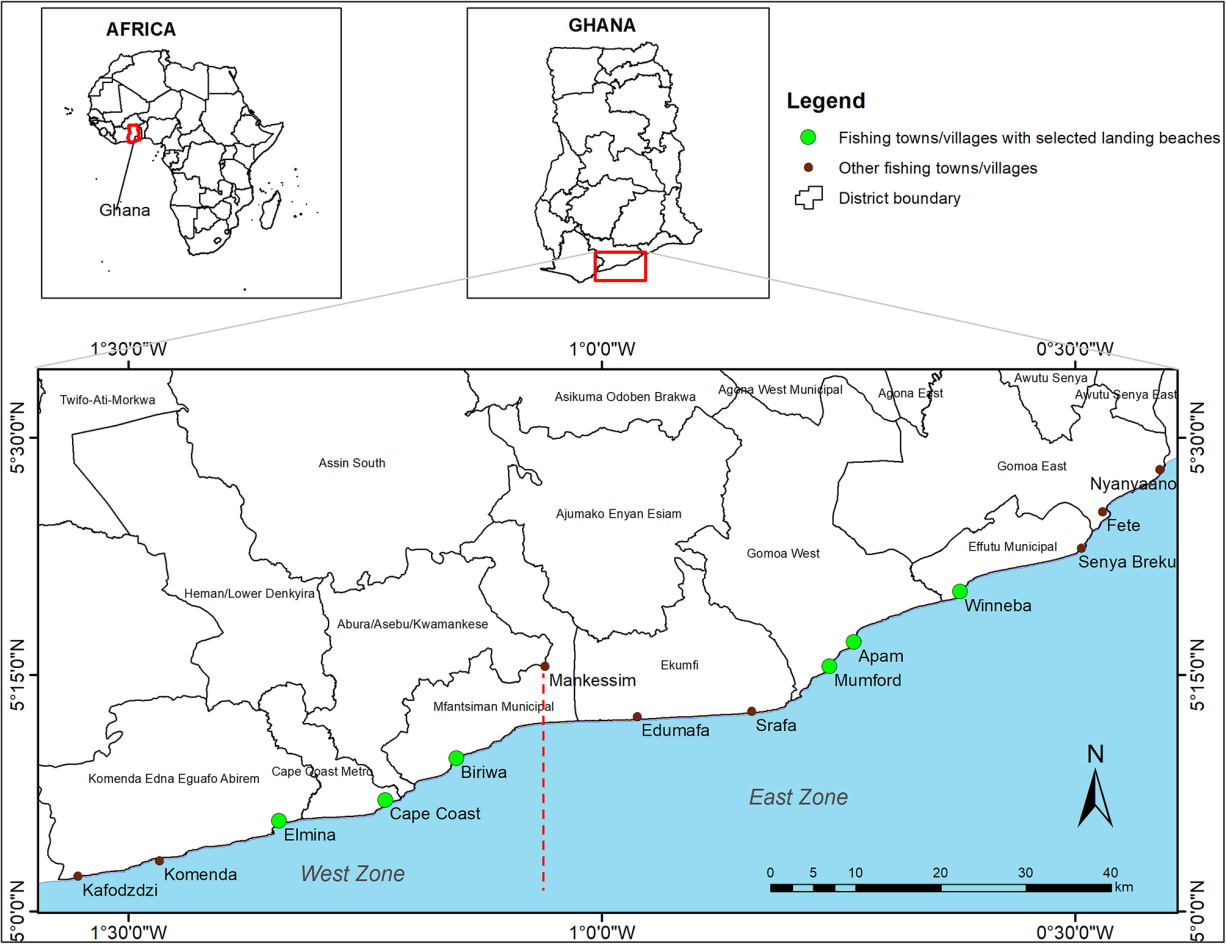
Study area showing locations of the six fish landing beaches selected for the study.

In the context of this study, “fishers” is used for both genders while “fishermen” and “fish processors” refer to men and women respectively. Categorisation and selection of landing beaches were based on the reported population of fishermen in the 2016 Ghana Canoe Frame Survey^5^. Using the estimated population of a minimum of 30 and maximum of 2,200 fishermen in the region, the landing beaches were categorised by terciles into low (30–700), moderate (701–1400) and highly (1401–2200) populated. To ensure a balance of spatial distribution of study locations across the region, the coastline was bisected into East and West zones (Fig. 1). One each of low, moderate and highly populated landing beaches were selected in each zone based on, firstly, location with the highest number of fishermen within the category, and secondly for the low population beaches, and finally based on the knowledge of the researchers on the existence of active fishing at the location. Using these criteria, the landing beaches selected were Winneba ‘Ayipei’ (high), Apam ‘Main’ (moderate) and Mumford ‘Main’ (low) in the East zone, and Elmina ‘Main’ (high), Biriwa ‘Abaka Ekyir’ (moderate) and Cape Coast ‘Abrofo Mpoano’ (low) in the West zone.

### UAV deployment and capturing of aerial imagery

An unmanned aerial vehicle, UAV–DJI Phantom 4, equipped with propeller guards (to prevent direct contact with any obstacle including flying animals) was deployed at the six landing beaches to capture aerial photos on different days from 10th April to 23rd April 2020. Drone deployment was carried out between 08:00 and 09:00 GMT since fish trade at the landing beaches usually spans the hours of 08:00 to 16:00 GMT with most vibrancy in the mornings. The flight missions were pre-planned and executed using DroneDeploy v.2.66.2 on an iPad mini 4. Several pre-flight tests informed the appropriate flight altitude of 60 m based on easy location of humans in the images, obscured identities in the overhead images, and safe height to avoid collision with any obstacles. The resolution of images captured was 1.8 cm. UAV flights were coordinated by pilots certified by the Ghana Civil Aviation Authority.

### Point data (human locations) extraction and measurement of nearest-neighbour distances

**Figure 2:**
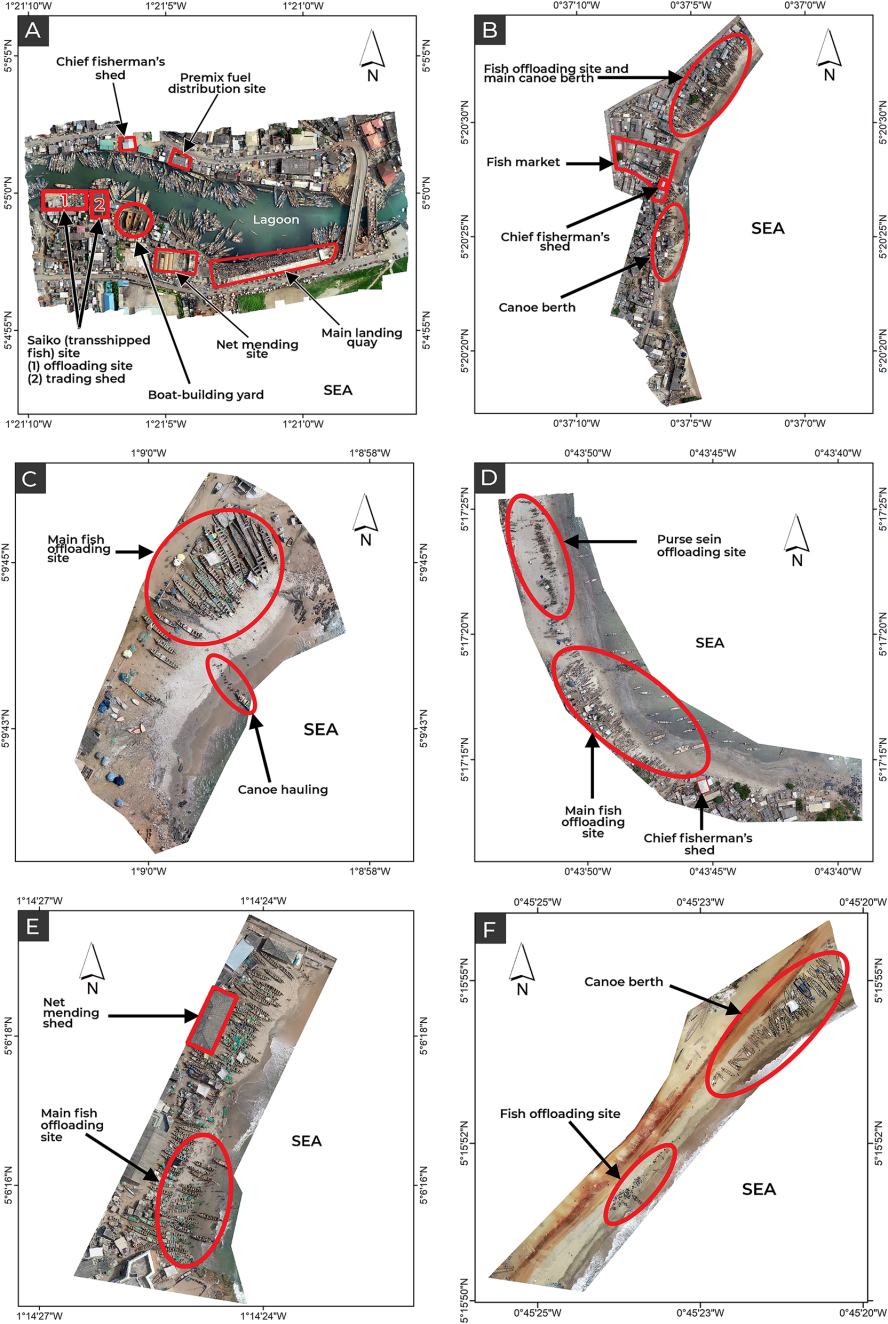
Orthomosaic images of the (**A**) Elmina ‘Main’, (**B**) Winneba ‘Ayipei’, (**C**) Biriwa ‘Abaka Ekyir’, (**D**) Apam ‘Main’, (**E**) Cape Coast ‘Abrofo Mpoano’, and (**F**) Mumford ‘Main’ landing beaches with artisanal fishing-related features marked.

The aerial photos obtained were uploaded into Agisoft Metashape Professional v.1.5.3.8407 and processed into seamless orthomosaic images, and identifiable features related to artisanal fishing at the various landing beaches marked (Fig. 2). The locations of people in orthomosaic images were manually extracted as point data in ESRI ArcMap v.10.3 using the editor tool. From the point data, the distance from each point to the nearest other point, that is the nearest-neighbour distance (NND), was measured for all individuals present in each of the six landing beaches in this study.

The median distances were compared to the WHO and Center for Disease Control (CDC) standards on physical (social) distancing. The term social distancing is used more frequently in this article due to its usage in common parlance. The application of UAV in a way that subjects are not identifiable and does not link information collected to the subjects qualifies the study for exempt human subject research (HSR)^17^, thus ethical clearance was issued by the Institutional Review Board (IRB) of the University of Cape Coast for the study. It is noteworthy, however, that aerial images from the UAV flights were unable to detect human presence at sheltered areas of the landing beaches, and as such there is likely an underestimation of proximity particularly in areas where fish trade occurs under sheds. Minor errors are also anticipated through the multiple capture of moving objects (persons) at margins of images. In addition to the imaging, hand washing facilities that have been placed at the landing beaches were manually counted and their geo-locations recorded with handheld Garmin GPS (Garmin GPSMAP 64s).

### Focus group discussions

The Chief Fishermen of all selected landing beaches were informed of the study through telephone calls, and their consent sought for the participation of their community in AUV surveys and associated stakeholder interviews. To engage fishers and industry participants in the study to understand their knowledge of COVID-19, perception of risks, and opinions on plausible solutions, we initially planned to conduct key informant interviews by telephone. These were, however, unsuccessful, and the strategy was revised to conduct brief focus group discussions (FGDs) in three communities. Two FGDs of not more than three key informants were conducted in each of the communities, ensuring strict adherence to the WHO’s protocols on social distancing, hand washing and wearing of face masks. For each landing beach, one FGD targeted fishermen (constituted of Chief Fisherman, a canoe owner and a fisherman) and another, the fish processors (the queen fish processor and a fish processor) to achieve disaggregation by gender of participants. These groups are the key stakeholders of the artisanal fisheries production value chain in Ghana. The discussions were recorded for summative analyses on the issues, and the results were later validated with the participants.

### Data analyses

Density and distance-based methods of point pattern analysis techniques^18^ were used to analyse the location of people (point data) in determining the hotspots of observation at the landing beaches based on quadrat density and Kernel density. All imagery analyses were carried out in the R-programme with the “sf”, “raster”, “sp”, “spatstat” and “maptools” packages. Quadrats of 2 m^2^ grids were employed, assuming 2 m as appropriate COVID-19 social distancing protocol. Further, homogeneity of locations of people at the landing beaches was tested with a non-parametric Chi-squared test of Complete Spatial Randomness (CRS) with the assumption that point process was Poisson Process (See Supplementary Table S1). Nearest-neighbour distance (NND) and the cumulative distribution function (G-function) of the NND, i.e. G(r), were used to compute the shortest distance from each person’s location to the nearest other person. The significance of any departures from Complete Spatial Randomness (either clustering or regularity) was evaluated using simulated “confidence envelopes”.

The means (± S.E.) of Log_10_(NND) for the landing beaches were compared using one-way ANOVA in Minitab 19.1. Independent measurements of NNDs were treated as repeated responses (response) for each landing beach (factor) and landing beach category (factor). Specific differences between landing beaches and categories were identified using Tukey HSD as post-hoc test. The data transformation to Log_10_(x) was applied following normality (Kolmogorov–Smirnov) and homogeneity (Cochran’s) tests. Median was, however, reported as the central tendency for NND due to the large proportion of outliers and skewness in the data (see Fig. 6). The level of significance and confidence level for all inferential statistics in the study were set to 0.05 and 95% respectively.

Analyses of information from the focus group discussions were guided by an inductive content analysis approach^19^. Responses were organized into main sections similar to the sections in the instrument, and general themes that emerged were developed after which sub-themes were created and assigned codes. Views and experiences of the fishers on the COVID-19 subject were identified under sub-themes to aid comparison, and final analyses were summarised and tabulated into a matrix of thematic issues and summary responses.

### Ethics declarations

Ethical approval for this study was given by the Institutional Review Board of the University of Cape Coast, all procedures followed the required ethical (Belmont) guidelines, and informed consent was obtained from all participants.

## Results

### Human densities at the landing beaches and potential clustering hotspots

**Figure 3:**
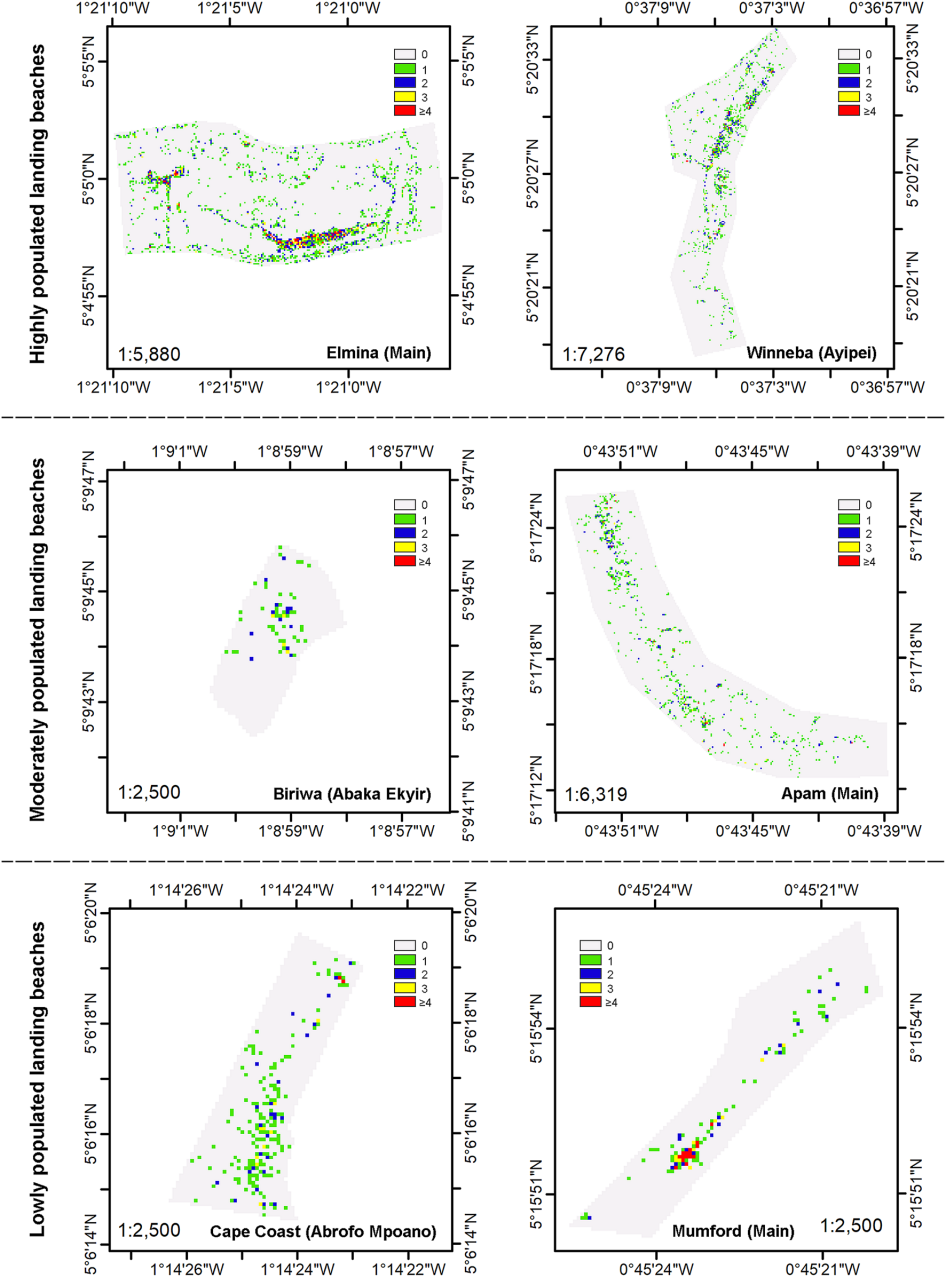
Quadrat density plots of humans at the six surveyed beaches (legend represents number of humans occurring in 2 m grid).

From the UAV imagery, a total of 2,890 individuals were recorded at the Elmina ‘Main’ landing beach, 1,632 at Winneba ‘Ayipei’, 1,053 at Apam ‘Main’, 236 at Cape Coast ‘Abrofo Mpoano’ and 209 at Mumford ‘Main’, with Biriwa ‘Abaka Ekyir’ having the lowest of 79 people. Quadrat density analyses of the point data (Fig. 3) showed the occurrence of humans across the length of all six landing beaches with noticeable aggregation at certain areas of each landing beach. In these clusters, concentration of 3 to 4 persons within 2 m^2^ grids as indication of crowding were prominently observed at Elmina and Mumford, considerably at Winneba, Apam and Cape Coast, and sparingly at Biriwa.

**Figure 4:**
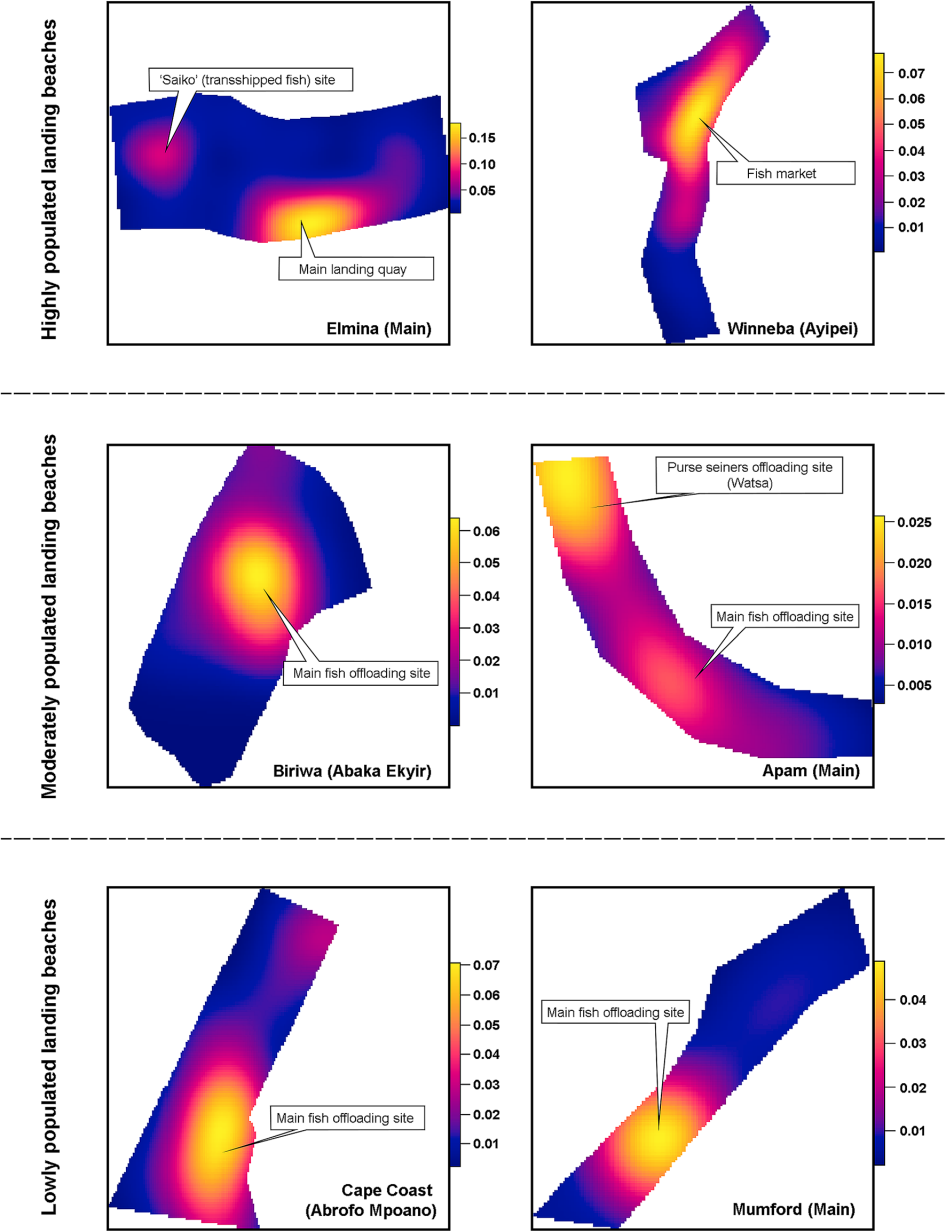
Kernel density for point pattern showing landing beach-specific hotspots for potential spread of infectious disease. Scale represents Kernel Function; independent for each landing beach and not for comparing landing beaches).

Results of the cumulative distribution function (G-function—see supplementary Fig. S1) further buttresses the occurrence of clustering at the beaches; clustering occurs when the observed G(r) curve is above the theoretical G(r) curve [i.e. G(r)obs > G(r)theo]. Hotspots in the Kernel density plot (Fig. 4) shows the specific locations of clustering at each landing beach, which are the potential hotspots for fast spread of COVID-19 in the communities in case of an outbreak. Generally, the clusters occurred at the main fish offloading sites at the landing beaches and imperceptibly at the canoe berths and net mending areas. At Elmina, two potential hotspots were observed, the first and most prominent being the main fish landing quay which incorporates a fish market, and the second occurring at the transhipped fish (locally known as “Saiko” fishing) offloading site. Similar dual hotspots were observed at Apam where the offloading site for the purse seine “*watsa*” fishers had even more intense clustering than the main offloading site. Like the Elmina fish landing quay, Winneba ‘Ayipei’ landing beach which also has a fish market integrated into the beach had the hotspot of clustering occurring within the market enclave.

### Physical distancing at the landing beaches

**Figure 5:**
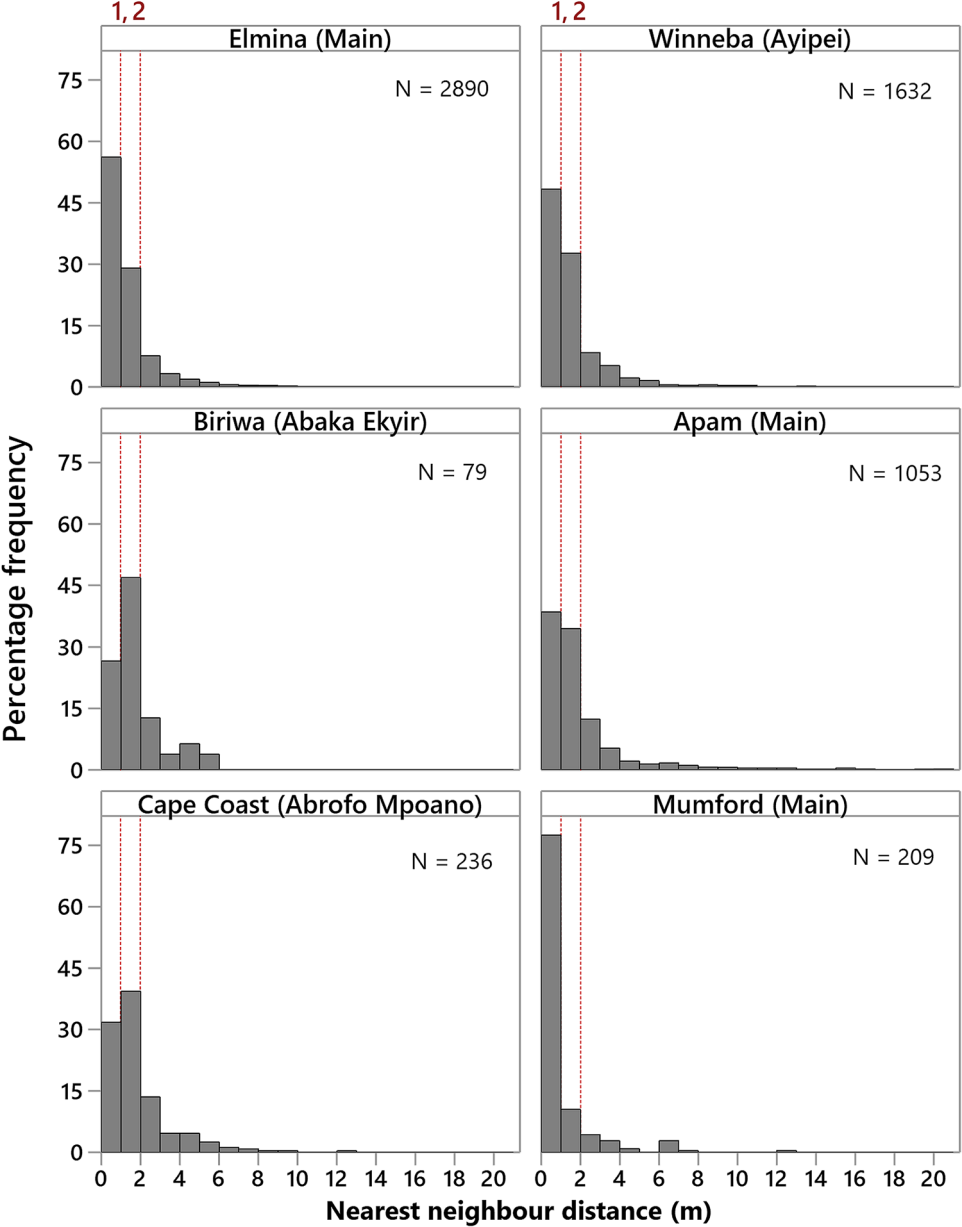
Nearest-neighbour distance at the six landing beaches (dotted vertical lines represent WHO reference of 1 m and CDC reference of 2 m minimum physical distance).

In all, 2,890, 1,632, 79, 1,053, 236 and 209 independent measurements of closest distance between persons (NNDs), in metres, were obtained at Elmina ‘Main’, Winneba ‘Ayipei’, Biriwa ‘Abaka Ekyir’, Apam ‘Main’, Cape Coast ‘Abrofo Mpoano’, and Mumford ‘Main’, respectively. Over 70% of the distances between persons recorded at each landing beach were less than 2 m from the nearest-neighbour (Fig. 5). Of a serious concern is the finding that the largest proportion of persons at the Elmina (56.06%), Winneba (48.22%), Apam (38.46%) and Mumford (77.51%) landing beaches were at distances less than the WHO recommended minimum of 1 m from their nearest-neighbour.

**Figure 6:**
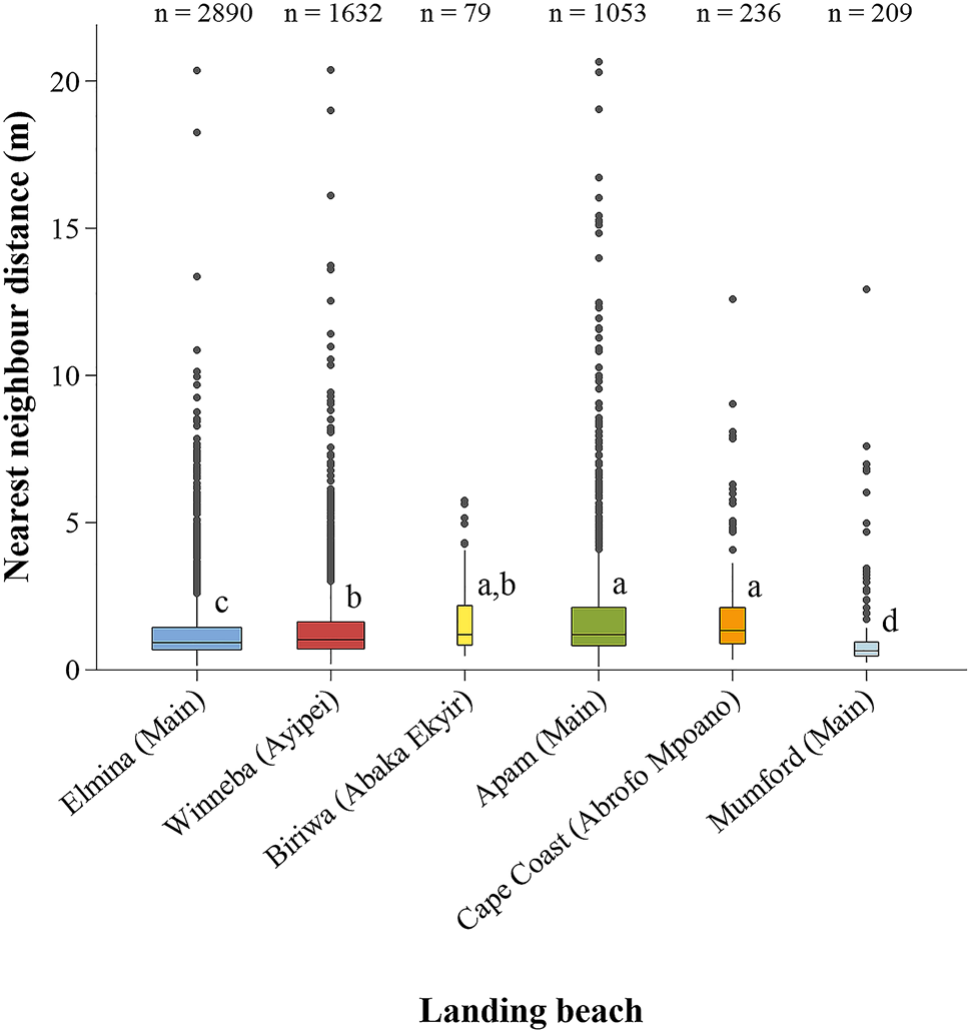
Distribution of nearest-neighbour distances (NND) at the various fish landing beaches (n = number of observations/distances). Alphabets represent significant differences (one-way ANOVA) in mean ± S.E. of Log_10_(NND) among the landing beaches; Groups that do not share a letter are significantly different.

Further, box plots of NND observations at the various landing beaches (Fig. 6) indicate median NNDs of less than 1.4 m for Winneba, Biriwa, Apam and Cape Coast whereas median NNDs below 1.0 m were recorded at Elmina and Mumford. Essentially this underscores the undermining of social distancing as a COVID-19 prevention protocol at the fish landing beaches. The situation was accentuated at Mumford, which had the most huddled individuals (median NND = 0.65 m). Although there was a significant difference (F = 52.56; *P* = 0.000) in NND among the categories of landing beaches, there was no sequential order from lowly, through moderately to highly populated categories of landing beaches in this study. Lowly and highly populated landing beaches were statistically similar (F = 46.79; *P* = 0.000) in NND. One-way ANOVA of Log_10_(NND) showed significant differences in crowding among the six landing beaches (F = 46.79; *P* = 0.000). Notably, Biriwa, Apam and Cape Coast had similar crowding situations comparing means of Log_10_ (NND) simultaneously (*P* > 0.05). The situation at Winneba was also similar to Biriwa (*P* = 0.000).

### Summary of outcomes from the focus group discussions

Feedback from the FGDs (Table 1) showed that fishers were aware of COVID-19, with some sensitisation being carried in the fishing villages by Municipal Assemblies and community opinion leaders through community radio stations and other information systems. Measures such as hand-washing facilities had been provided at the landing beaches to improve hygiene and mitigate spread of the disease. During the field visits, seven hand-washing stations were counted at Elmina, four at Winneba, two each at Cape Coast and Apam, and one each at Biriwa and Mumford. While the fish processors were of the view that fishers were not cooperative in observing social distancing and other measures, the fishermen indicated that fishers were cooperative. To the fishermen, the most difficult period to maintain appropriate distance from others was during offloading fish and hauling their canoes to berth while for the women, their difficulty was during purchases of offloaded fish and selling at the landing beach. The pandemic had negatively impacted the fishermen through restrictions on their movements to neighbouring West African countries to fish, and they had additionally reduced the crew size in the canoes to decrease crowding, thereby reducing the labour force, catch and income which had in turn affected sales and income of the women. The fishers mentioned logistical and financial support was needed to help prevent and mitigate the spread and impact of COVID-19 at the landing beaches, suggesting the need for engaging law enforcement agencies to ensure compliance with social distancing at the landing beaches.

**Table 1:** Summary of FGDs conducted at the landing beaches.

Thematic issue	Summary of response	
	Fishermen	Fish processors
Awareness of COVID-19	Aware through radio and TV	
Mode of sensitization in the community	Community radio and information centres, posters	Community radio and information van
Measures in place to mitigate spread	Provided hand-washing facilities and advising on social distancing	Provided hand washing facilities and advising on social distancing and personal hygiene
Cooperation of fishers to mitigation measures	Very cooperative and adhering to the measures	Not too cooperative
Most difficult period to maintain appropriate distance from others	Offloading fish and hauling their canoes to berth after landing	Buying offloaded fish and selling fish at the beach
Impact of COVID-19 on fishing activities	Restrictions especially to other countries for fishing, low fish catch due to reduction in fishing crew, reduced incomes	Reduction in sales due to low fish catch, reduced income
Roles of Fisher Leadership in mitigating measures	Provided some of the hand-washing facilities, have been sensitising fishers	Have been sensitizing fishers about the disease
Role of Fisheries Commission and Municipal Assemblies in helping prevent COVID-19	Municipal Assemblies carry out sensitization, have provided some of the hand-washing facilities and conducted mass testing at some landing beaches awaiting results. Marine police of the Fisheries enforcement unit also carried out some education	
Support needed to enhance safety of fishers	Financial support, more hand-washing facilities, hand sanitizers, face masks, and food supply	Attitudinal change by fishers, provision of sanitizers, face masks, food supply and financial support
How to effectively adhere to social distancing during fishing activities	Properly organise the process of buying fish at the beaches, intensify sensitization and use law enforcement agencies	Ensure compliance through use of law enforcement agencies

## Discussion

From the onset of COVID-19, the pandemic has been projected to take a significant toll on global fisheries, with predictions of potentially negative consequences for the livelihoods and incomes of the multitudes of small-scale fishers in developing countries^10,11^. Although it is seemingly early to meaningfully appreciate the effects of the disease on fisheries, at the time of this study, reports had started trickling in on its impacts on small scale fisheries^20^. Changing consumer demands, market access and border restrictions among others, are predicted to be the major drivers of the impact of COVID-19 on fisheries^10,11^. Aside from these, there is heightened apprehension that fishing communities are at high risk of COVID-19 due to the migratory and clustering behaviour of fishers, making the fishing communities potential “hotspots” for rapid spread of the disease^11^.

Considering that the disease spreads mainly through aerosolisation during person-to-person contact^9^, it is critical to ensure that fishers adhere to the protocols on preventive measures while carrying out their activities. Central to this study is the adherence to the protocol on social distancing, prescribed by WHO as a minimum of 1 m^8^ and the CDC as minimum of 6 ft (≈ 1.8 m)^9^ physical distance required to be kept from others in avoidance of contracting the virus.

Results of this study have shown that huddling and social distancing remain a major challenge at fish landing beaches in the Central Region of Ghana in the face of COVID-19. Clustering of fishers at densities up to four people within a 2 m^2^ area at most of the landing beaches, and over 70% of the people across landing beaches occurring at less than 2 m from their nearest-neighbours importantly underscores the need to devote attention to the crowding situation at the beaches. Another worrying situation is the occurrence of a substantial proportion of people at Elmina (≈ 56%), Winneba (≈ 48%), Apam (≈ 39%) and Mumford (≈ 78%) at a distance of less than 1 m from their nearest-neighbours which provides ample evidence of inability of fishers to voluntarily adhere to both WHO and CDC social distancing protocols despite being aware of the disease and the continuous sensitisation. Concerningly, these findings contradict the views of the fishermen that fishers are very cooperative in observing the mitigation measures, but supports the opinion of the women fish processors that fishers are not too cooperative with the measures. Heat maps from the Kernel density plot further point to the fish offloading sites and fish trading areas at the landing beaches as the hotspots for clustering, hence, the potential areas for escalating the spread of the disease. This corroborates the feedback from the fishermen that the most difficult period to maintain appropriate distance from others was during offloading and hauling of their canoes, and in the case of the fish processors, when purchasing offloaded fish and trading at the landing beach. Specific measures targeting the various activities at the landing beaches will therefore be required to mitigate the spread of the disease as articulated in latter sections of the discussion.

Hypothetically, it was expected that the highly populated landing beaches documented in the canoe frame survey^5^, may have higher incidence of crowding and therefore increased risk of people contracting the COVID-19 disease through huddling. However, the absence of a correlation between huddling and landing beach population categories (i.e. low, moderate and highly populated) reinforces that it is the nature of the activities engaged in, rather than population size, that drives risk. For instance, Biriwa ‘Abaka Ekyir’ which fell within the moderately populated category with 246 fishermen^5^, had relatively low activity compared to the “lowly populated” Cape Coast ‘Abrofo Mpoano’ and Mumford ‘Main’. The contrast between the intensity of activities observed at these landing beaches at the time of field data collection and the expected intensity based on available data^5^, could mainly be resulting from low fish catch in the lean fishing season [i.e. November to May^3^]. During this fishing season, fishers migrate from rural landing sites to urban fish landing beaches to access cosmopolitan fish markets such as that of Cape Coast and Elmina. In addition, Mumford ‘Main’, a low population category landing site, which in this study had about one-fourteenth the number of people observed at Elmina ‘Main’ (highest number of people in this study), was estimated to be the most crowded amongst the six (median NND = 0.65 m). The mode of business transaction at the landing site is a major contributing factor here. Mumford ‘Main’ is a sandy beach landing site with no sheds, forcing fish traders (including middle-men) and processors to congregate in proximity in anticipation of fish offloads and direct sales from canoes. This brings to the fore the need for more industry specific analysis of worker functions and work environment context in making decisions on interventions in preventing the spread of COVID-19 and potentially other similar pandemics.

Our research suggests that fish landing sites across the coast, irrespective of size and population density, would require similar urgency and attention towards the prevention of the spread of highly infectious diseases like COVID-19. In this regard, such rapid, remote, and real-time assessments as used in this study could be a transformative tool to facilitate immediate informed response. The undesirable clustering at the landing beaches in the Central Region is akin to the situation at most of the nearly 300 coastal landing beaches in the country. Unfortunately, stringent measures instituted by the Metropolitan, Municipal and District Assemblies (MMDAs) to enforce appropriate physical distancing focused on agricultural markets has been at the neglect of the fish landing beaches. So stringent have these measures been, that non-compliant agricultural markets have been shut down^21^. Contrary to the agricultural markets, no fisher was spotted wearing a face mask at the landing beaches during the field visit. As noted from the field and focus group discussions, the leadership of the fishers acquired a small number of hand-washing facilities to improve hygiene at the beaches, and the respective MMDAs had also provided additional hygienic facilities coupled with occasional sensitisation, including carrying out mass testing at areas such as Winneba. Beyond these localised efforts, the Ministry of Fisheries and Aquaculture Development distributed a significant number of hand-washing facilities and face masks to most fishing communities along the coast approximately one month after our field survey. However, a recent assessment of all 300 landing beaches revealed that the hand-washing facilities were functional at less than ten landing beaches, and about 96% of the landing sites had only a few or none of the people wearing face masks^24^. Given the communal nature of artisanal fishing, low literacy rate of fishers^5^ and the illusion that the proximity of fishers to saline water renders them immune to COVID-19^15^, it is perhaps unrealistic to expect fishers to voluntarily comply with social distancing with the level of support currently offered.

Based on observation of direct behaviours and attitudes expressed in FGDs, our pilot study demonstrated the inadequacy of current provisions to reduce risk of COVID-19 transmission in the focus sites. A targeted prevention strategy is importantly needed for coastal communities as three of the four coastal regions are currently among the top five regions with the highest COVID-19 cases out of the sixteen regions in Ghana. These coastal regions are the Greater Accra Region (24,826), Western Region (2974) and Central Region (1931), which together represent 62% of the total confirmed cases of 47,690 as of Sunday, October 25, 2020^16^. A two-pronged approach would be suitable for reducing the risk of fishers; first by implementing targeted landing beach-wide interventions and secondly, workplace or activity-specific measures. At the landing beach level, deploying strategies specific to each fishing community within which the landing beach is located will be helpful. Vacant land spaces adjacent to landing beaches (e.g. in Elmina) could be repurposed to provide additional space for fish trading activities in order to minimise crowding in the main offloading sites and keep fish mongers and buyers spread out. Where there are no vacant spaces, regulating the number of canoes to fish in a day, staggering landing times, running a shift system for the fishers, and designating entry and exit points could be considered. These will limit the number of people and personal contact at the beaches. Though this could deepen the economic impact on the fishers, it has generally been successful in agricultural markets. Economic and other livelihood support for vulnerable fishers affected by such measures could be considered to ameliorate the impacts on their households.

The workplace or activity-specific measures should target offloading of fish, hauling of canoes and nets, purchasing and marketing, as well as processing of fish. In carrying out labour-intensive activities such as manual hauling of canoes and offloading of fish, which make it practically difficult for fishers to maintain appropriate physical distances from others, wearing of face masks by the fishers should be adopted as primary prevention protocol as prescribed by the WHO. This is particularly important as the process of hauling canoes and nets by artisanal fishers in Ghana are characterised by the culture of chanting work-songs accompanied by instructional shouts through which aerosols could be transmitted and possibly spread the disease if present. The wearing of face masks should also be extended to the fish purchasing, marketing and processing activities as they involve yelling for customers and active bargaining.

Considering that regular human and material contacts are inevitable during fishing activities due to the lack of physical distancing demonstrated in this study, frequent washing and disinfection of hands and all fishing equipment are imperative. From the FDGs, there were indications of some provisions of hand washing facilities at the landing beaches. Nonetheless, some of the facilities were observed without water, soap and other needed consumables during the field visit. Adding to the existing hand washing facilities and importantly ensuring frequent supply of these consumables including hand sanitizers are critical to enhancing COVID-19 hygienic practices at the landing beaches. Although very little fish in the artisanal fish value chain goes through cold storage, cold-chain transportation of food including fish is reportedly an important way of spreading the virus^25^. Therefore, instituting modalities for disinfecting frozen fish at imported fish outlets and the transhipped fish (Saiko) landing sites is also very critical as both the transhipped fish from industrial trawlers and imported fish end up mainly with fish processors along the coast.

For a rather lasting intervention, the approach of behavioural change communications could be used as a strategy to achieve voluntary compliance to physical distancing by the fishers. By disabusing the fisherfolk of their misconception about the disease, presenting customised COVID-19-related information relevant to their trade, and supporting with the specific workable practices discussed in this paper, fishers could understand better the need for social distancing and practice same. Direct feedback to communities and the local MMDAs from rapid appraisal such as that conducted here may be instrumental in gaining recognition that current practices are not adequate and reinforcing the areas of high risk. A coordinated effort of MMDAs and the Fisheries Commission is crucial in addressing the situation at the landing beaches, and funds for the intervention could be collaboratively drawn from the District Assemblies Common Fund, the Fisheries Development Fund and the Government of Ghana’s COVID-19 intervention fund. Even though studies have suggested that SARS-CoV-2 thrives better and possibly spreads faster in the mid-to-higher latitudes than in the lower latitudes due to optimal temperature and humidity for its spread in these climatic zones^26^ which may account for the lower number of cases recorded in Ghana and other parts of Africa, it is important not to relent on ensuring adherence to the preventive protocols in the face of rising number of cases in the country.

Lastly, the safe utilisation of UAV in this work, which qualifies for exempt UAV-Human Subject Research^17,22^, advances drone applications in fisheries research^23^ through the application of UAV in assessing clustering related to the fish trade in a developing country towards curtailing the spread of infectious diseases. Among others, the demonstration in this study could serve as a reference for rapid behavioural prognostics in fisheries, especially during diseases of the nature of COVID-19.

## Conclusion

Unmanned Aerial Vehicle (UAV) technology was successfully used to rapidly and efficiently assess the risk to artisanal fishers from the pandemic using social distancing as a proxy: an approach that can be easily and quickly scaled up for broader application. The findings of the study have demonstrated that clustering and social distancing remains a challenge at fish landing beaches in the Central Region of Ghana in the face of growing cases of COVID-19 in the country. A significant level of clustering occurred within distances of less than 1 m, indicating the inability of fishers to voluntarily adhere to both WHO and CDC social distancing protocols irrespective of their awareness of the disease. Enforcement at these beaches was also of great concern as no designated measures had been put in place to ensure supervised compliance. The risk of artisanal fishers to the COVID-19 disease from clustering was independent of the size of landing beach. Therefore, an urgent concerted effort across relevant health and fisheries governance institutions is required to institute radical prevention and mitigation measures. This effort should be focused primarily on implementing targeted landing beach-wide interventions on one hand and workplace or activity-specific measures on the other hand. Specifically, the judicious use of land spaces adjacent to landing beaches could present an opportunity for interspersing fish trading activities to reduce clustering. It is also recommended that fishers be compelled to adhere to wearing face masks during fish offloading, canoe hauling, and fish trading, when it is impractical to observe social distancing. It is noteworthy that this assessment was conducted during the lean fishing season and crowding situation could worsen in the main or bumper fishing season (July – September) posing an even greater risk should the disease persist. These findings provide a point of entry for government agencies, donors and NGOs in identifying potential support that could be provided to the landing beaches to reduce the risk to fishers, their families and community to the novel coronavirus disease.

## Supplementary Information

Supplementary Infomation.

## Data Availability

The datasets generated and used during this study are available on the Mendeley's free data repository platform (https://data.mendeley.com/datasets/2s6x25xsrd/1) with the 10.17632/2s6x25xsrd.1
