# Supplementary material for: Physical distancing and risk of COVID-19 in small-scale fisheries: a remote sensing assessment in coastal Ghana
Source: Sci Rep. 2020 Dec 29;10:22407. doi: 10.1038/s41598-020-79898-4 (PMC7772332; doi:10.1038/s41598-020-79898-4)
Supplement: Supplementary file 1 — Supplementary Infomation. [file 41598_2020_79898_MOESM1_ESM.pdf]

Physical distancing and risk of COVID-19 in small-scale fisheries: A remote sensing assessment in coastal Ghana

Isaac Okyere, Ernest O. Chuku, Bernard Ekumah, Donatus B. Angnuureng, Justice K. Boakye-Appiah, David J. Mills, Raymond Babanawo, Noble K. Asare, Denis W. Aheto, Brian Crawford

Supplementary Data

***Supplementary Table 1*: Chi-squared test of Complete Spatial Randomness (CSR) using quadrat counts**

Fishing village (landing beach)	Degrees of Freedom	X^2	P-Value
Elmina (Main)	24	2678.2	<0.0001
Winneba (Ayipei)	19	933.74	<0.0001
Biriwa (Abaka Ekyir)	20	147.86	<0.0001
Apam (Main)	15	447.64	<0.0001
Cape Coast (Abrofo Mpoano)	17	263.77	<0.0001
Mumford (Main)	13	539.38	<0.0001

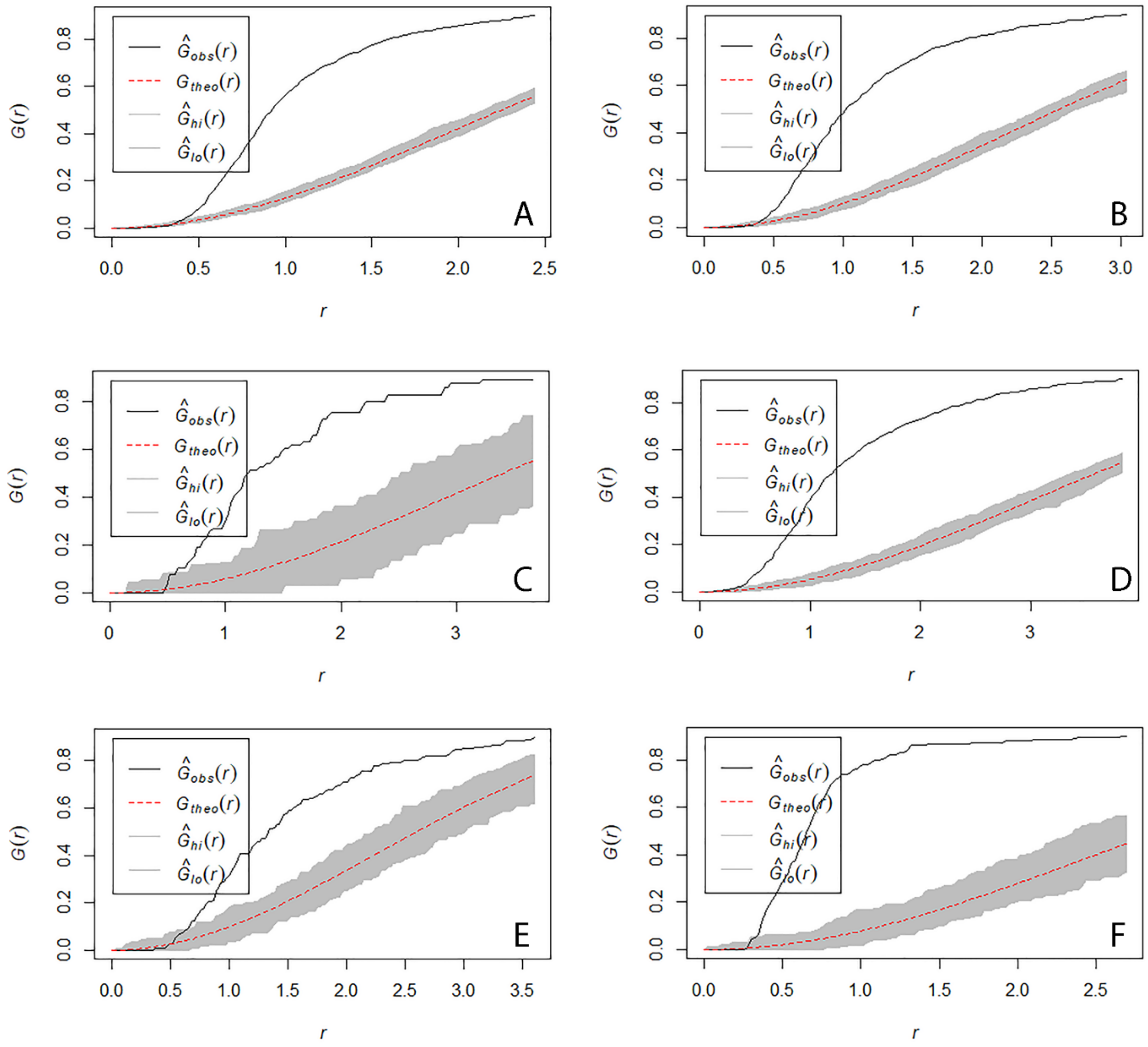

***Supplementary Figure 1*:** Cumulative Distribution of Nearest-Neighbour Distances G(r) Plots for the Landing Beaches
